# Synthesis of Silver Nanoparticles Mediated by Fungi: A Review

**DOI:** 10.3389/fbioe.2019.00287

**Published:** 2019-10-22

**Authors:** Mariana Guilger-Casagrande, Renata de Lima

**Affiliations:** Laboratory for Evaluation of the Bioactivity and Toxicology of Nanomaterials, University of Sorocaba, Sorocaba, Brazil

**Keywords:** biogenic synthesis, silver nanoparticles, fungi, biological activity, biomolecules

## Abstract

The use of fungi as reducing and stabilizing agents in the biogenic synthesis of silver nanoparticles is attractive due to the production of large quantities of proteins, high yields, easy handling, and low toxicity of the residues. Furthermore, this synthesis process coats the nanoparticles with biomolecules derived from the fungus, which can improve stability and may confer biological activity. The aim of this review is to describe studies in which silver nanoparticles were synthesized using fungi as reducing agents, discussing the mechanisms and optimization of the synthesis, as well as the applications. The literature shows that various species of fungus have potential for use in biogenic synthesis, enabling the production of nanoparticles with different characteristics, considering aspects such as their size, surface charge, and morphology. The synthesis mechanisms have not yet been fully elucidated, although it is believed that fungal biomolecules are mainly responsible for the process. The synthesis can be optimized by adjusting parameters such as temperature, pH, silver precursor concentration, biomass amount, and fungus cultivation time. Silver nanoparticles synthesized using fungi enable the control of pathogens, with low toxicity and good biocompatibility. These findings open perspectives for future investigations concerning the use of these nanoparticles as antimicrobials in the areas of health and agriculture.

## Introduction

Among the different types of metallic nanoparticles, silver nanoparticles can be highlighted for their broad-spectrum antimicrobial potential (Prabhu and Poulose, 2012; Rai et al., 2014; Gupta et al., 2017; Loo et al., 2018). These nanoparticles adhere to the cell walls and membranes of microorganisms and may reach the cell interior. They damage the cellular structures, induce the production of reactive oxygen species, and alter the mechanisms of signal transduction (Kim et al., 2011; Dakal et al., 2016). Several studies report applications in which good results have been obtained using silver nanoparticles for the control of pathogenic microorganisms in the areas of health and agriculture (Kim et al., 2012; Mishra and Singh, 2015; Burduşel et al., 2018).

The commonest method used to produce silver nanoparticles is chemical synthesis, employing reagents whose function is to reduce the silver ions and stabilize the nanoparticles. These reagents are toxic and can present risks to health and the environment (Ahmed et al., 2016; Zhang et al., 2016), which has led to increasing interest in biogenic synthesis methods. Such processes enable nanoparticles to be obtained that present lower toxicity, better physicochemical characteristics, and higher stability (Iravani et al., 2014).

Biogenic synthesis of nanoparticles can be performed using organisms such as bacteria, fungi, and plants, or the byproducts of their metabolism, which act as reducing and stabilizing agents (Durán et al., 2011). These nanoparticles are capped with biomolecules derived from the organism used in the synthesis, which can improve stability and may present biological activity (Ballotin et al., 2016). Biogenic synthesis is relatively simple, clean, sustainable, and economical, and provides greater biocompatibility in the uses of nanoparticles (Gholami-Shabani et al., 2014).

The synthesis of silver nanoparticles using bacteria involves cultivating the organisms in a suitable medium, followed by contact of the bacteria or their metabolites with a silver nitrate (AgNO3) solution (Singh et al., 2015). When the synthesis is performed using plants, aqueous extracts are prepared from the leaves, stems, or roots, followed by the addition of silver nitrate solution (Rheder et al., 2018). The use of fungi to synthesize silver nanoparticles involves culturing the fungus on agar, followed by transfer to a liquid medium. The biomass produced is subsequently transferred to water for release of the compounds that act in the synthesis. After filtration, the biomass is discarded and silver nitrate is added to the filtrate (Costa Silva et al., 2017; Guilger et al., 2017; Mekkawy et al., 2017; Ottoni et al., 2017).

The purpose of this review is to provide an overview of the main published studies concerning the use of fungi for the biogenic synthesis of silver nanoparticles, as well as the applications of these materials in different areas. The synthesis mechanisms are discussed, together with methods to optimize the processes and the importance of cappings on the nanoparticles.

Given the problems caused by pathogenic microorganisms, there is a continuing search for more effective techniques for their control. The emergence of nanotechnology has led to increasing interest in the antimicrobial properties of silver nanoparticles and in exploring environmentally friendly ways in which they can be used most effectively.

## Biogenic Synthesis of Silver Nanoparticles

Most of the conventional methods used to produce nanoparticles have disadvantages such as the use of toxic chemicals and the generation of waste, which can cause environmental pollution (Iravani et al., 2014; Ahmed et al., 2016). Consequently, in recent years there has been increasing interest in eco-friendly synthesis methods. The methods involve the use of organisms including bacteria, fungi, and plants, which can reduce metal salts and enable the formation of nanoparticles that present the desired size and morphology (Azmath et al., 2016). The production of nanoparticles by biological reduction of metals is an option that can be considered clean, non-toxic, and environmentally acceptable (Banu and Balasubramanian, 2014b).

Fungi are attractive agents for biogenic synthesis of silver nanoparticles, because they offer high tolerance to metals and are easy to handle. They also secrete large quantities of extracellular proteins that contribute to the stability of the nanoparticles (Balaji et al., 2009; Du et al., 2015; Netala et al., 2016). Advantages of fungal cultures over bacterial systems are that they provide good biomass production and do not require additional steps to extract the filtrate (Gade et al., 2008). Compared to synthesis using plants, the mycelial mass of fungi is more resistant to agitation and pressure, so it is more suitable for large-scale syntheses (Velusamy et al., 2016). Furthermore, by adjusting culture conditions such as time, temperature, pH, and quantity of biomass, among others, it is possible to manipulate the metabolism of fungi so as to obtain nanoparticles with the desired characteristics, such as specific size and morphology (Zielonka and Klimek-Ochab, 2017).

### Biogenic Synthesis of Silver Nanoparticles Mediated by Fungi

Fungi have excellent potential for the production of many compounds that can be used in different applications. Around 6,400 bioactive substances are known to be produced by microscopic filamentous fungi (ascomycetes and imperfect fungi) and other fungal species (Bérdy, 2005). These organisms are widely used as reducing and stabilizing agents, due to their heavy metal tolerance and capacity to internalize and bioaccumulate metals. Furthermore, fungi can be easily cultivated on a large scale (“nanofactories”) and can produce nanoparticles with controlled size and morphology (Gade et al., 2008; Ahluwalia et al., 2014; Azmath et al., 2016; Khan et al., 2017). Fungi have advantages over other microorganisms, in that they produce large quantities of proteins and enzymes, some of which can be used for the fast and sustainable synthesis of nanoparticles (Vahabi et al., 2011; Alghuthaymi et al., 2015).

The mechanism of biogenic synthesis of nanoparticles using fungi may be intracellular or extracellular. In the case of intracellular synthesis, the metal precursor is added to the mycelial culture and is internalized in the biomass. Consequently, extraction of the nanoparticles is required after the synthesis, employing chemical treatment, centrifugation, and filtration to disrupt the biomass and release the nanoparticles (Castro-Longoria et al., 2011; Rajput et al., 2016; Molnár et al., 2018). In extracellular synthesis, the metal precursor is added to the aqueous filtrate containing only the fungal biomolecules, resulting in the formation of free nanoparticles in the dispersion. This last method is most widely used, since no procedures are required to release the nanoparticles from the cells (Azmath et al., 2016; Sabri et al., 2016; Costa Silva et al., 2017; Gudikandula et al., 2017). Nonetheless, the nanoparticle dispersion must be purified in order to eliminate fungal residues and impurities, which can be achieved using methods such as simple filtration, membrane filtration, gel filtration, dialysis, and ultracentrifugation (Ashrafi et al., 2013; Qidwai et al., 2018; Yahyaei and Pourali, 2019).

## Synthesis Mechanisms

### How Does Extracellular Synthesis of Silver Nanoparticles by Fungi Occur?

Although many studies have been published concerning the biogenic synthesis of silver nanoparticles using fungi, the specific mechanisms involved have not yet been fully elucidated. It is known that extracellular synthesis of nanoparticles occurs according to reactions in which the enzymes present in the fungal filtrate act to reduce silver ions, producing elemental silver (Ag^0^) at a nanometric scale. After the reaction, the color of the filtrate changes and UV-visible spectroscopy can be used to observe surface plasmon resonance bands reflecting alteration of the optical properties of the material (Ahmad et al., 2003). The absorbance wavelengths of these bands vary in the range from 400 to 450 nm, with an absorbance peak at a longer wavelength indicating the presence of larger nanoparticles (Elamawi et al., 2018). The size depends on the synthesis conditions such as fungus species, temperature, pH, and dispersion medium, as well as the presence of cappings on the nanoparticles (Khandel and Shahi, 2018; Lee and Jun, 2019). The color of the dispersion is also directly related to the surface plasmon resonance, which varies according to the size and absorbance of the nanoparticles (Adeeyo and Odiyo, 2018; Bhangale et al., 2019; Lee and Jun, 2019).

**Figure 1:**
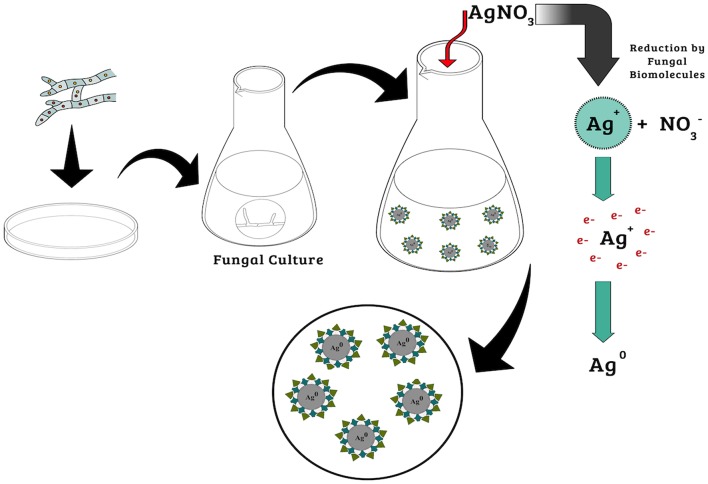
Mechanisms of biogenic synthesis of silver nanoparticles.

Many biomolecules can react with silver ions and act in the synthesis, such as those associated with the complex pathways involving electron transfer during the conversion of NADPH/NADH to NADP^+^/NAD^+^ (Thakkar et al., 2010; Gudikandula et al., 2017). Nicotinamide adenine dinucleotide (NADH) and NADH-dependent nitrate reductase enzymes are considered to be most important in the biogenic synthesis of metallic nanoparticles (Zomorodian et al., 2016; Baymiller et al., 2017; Figure 1).

In recent work, Hietzschold et al. (2019) showed that nanoparticle synthesis occurred by the action of NADPH, without any need for the nitrate reductase enzyme. This is particularly interesting, since it leads to the possibility of using different organisms for the synthesis of nanoparticles, without the necessary condition of reductase enzyme production. However, Durán et al. (2005) synthesized silver nanoparticles using *Fusarium oxysporum* and suggested that the reduction of silver ions was due to the action of the nitrate reductase enzyme and anthraquinones. In another study, using purified nitrate reductase and phytochelatins from the same fungus, it was found that extracellular NADPH-dependent nitrate reductase enzymes and quinones were responsible for the formation of nanoparticles (Kumar et al., 2007).

### Optimization of Silver Nanoparticles Synthesis

Although the synthesis of silver nanoparticles using fungi is simple and effective, the parameters employed in the procedure must be optimized in order to achieve good monodispersity, stability, and biocompatibility of the particles (Balakumaran et al., 2015). Given that a wide variety of fungi have potential for use in the synthesis, it is important to consider their individual characteristics and to optimize the synthesis conditions accordingly (Ottoni et al., 2017).

Parameters such as agitation, temperature, light, and culture and synthesis times differ depending on the fungus used and can also be adjusted in order to obtain the desired nanoparticle characteristics. Control of nanoparticle size and shape requires adjustment of the parameters used for both cultivation of the fungus and the synthesis process (Birla et al., 2013). Studies have found that changes in temperature, concentration of the metal precursor, pH, culture medium, and amount of biomass can be used to obtain nanoparticles with different physicochemical characteristics (Birla et al., 2013; Rajput et al., 2016; Saxena et al., 2016; Liang et al., 2017). Table 1 shows some studies in which silver nanoparticles were produced using different fungal species and synthesis conditions.

**Table 1:** Optimization of the synthesis of silver nanoparticles by fungi.

**Fungus**	**Type of parameter**	**Optimized conditions**	**Nanoparticle characteristics**	**References**
*Trichoderma harzianum*	Temperature and concentration of AgNO_3_	40°C 1 mM AgNO_3_	51.10 nm −17.19 mV Spherical	Ahluwalia et al., 2014
*Fusarium oxysporum*	Culture media, quantity of biomass, light intensity, pH, temperature, and concentration of AgNO_3_	MGYP 6 g of biomass 190.7 Lux and sun light pH 9 and 11 40 and 60°C 1.5 mM AgNO_3_	10–20 nm 30 mV Spherical	Birla et al., 2013
*Colleotrichum* sp. ALF2-6	Temperature and pH	50–80°C Alkaline pH	5–60 nm Myriad shapes	Azmath et al., 2016
*Aspergillus oryzae* (MTCC no. 1846)	Temperature, pH and concentration of AgNO_3_	90°C pH 10 1 mM AgNO_3_	7–27 nm Spherical	Phanjom and Ahmed, 2017
*Rhizopus stolonifer*	Temperature and concentration of AgNO_3_	40°C 10 mM AgNO_3_	2.86 nm Spherical	AbdelRahim et al., 2017
*Aspergillus fumigatus* BTCB10	Temperature, fungal culture age, quantity of biomass, pH, concentration of AgNO_3_, ratio of cell-free filtrate/silver nitrate	25°C 7 days of culture 7 g of biomass pH 6 1 mM AgNO_3_ Cell-free filtrate/silver nitrate 3:2	322.8 nm PDI 0.278 Spherical	Shahzad et al., 2019
*Fusarium oxysporum*	Temperature, quantity of biomass, pH and concentration of AgNO_3_,	50°C 11 g of biomass pH 6 10 mM AgNO_3_	5–13 nm Spherical	Husseiny et al., 2015
*Trichoderma viride*	Temperature	27°C	2–4 nm Spherical	Fayaz et al., 2009
*Isaria fumosorosea*	Temperature, pH and agitation	30°C pH 8.5 150 rpm	51.31–111.02 nm Spherical	Banu and Balasubramanian, 2014a
*Guignardia mangifera*	Temperature, quantity of biomass, pH and concentration of AgNO_3_	30°C 10 g of biomass pH 7 1 mM AgNO_3_	5–30 nm Spherical	Balakumaran et al., 2015
*Duddingtonia flagans*	Temperature and pH	60°C pH 10	30-409 nm −28,655 mV Spherical	Costa Silva et al., 2017
*Trichoderma longibrachiatum*	Temperature, quantity of biomass and agitation	28°C 10 g of biomass Without agitation	24.43 nm PDI 0.420 −19,7 mV Spherical	Elamawi et al., 2018
*Penicilliumpurpurogenum*	pH and concentration of AgNO_3_	pH 8 1 mM AgNO_3_	8–10 nm Spherical	Nayak et al., 2011
*Epicoccum nigrum*	Temperature, pH and concentration of AgNO_3_	55°C Alkaline pH 1 mM AgNO_3_	1–22 nm Spherical or near to spherical	Qian et al., 2013
*Penicillium oxalicum*	pH	pH 12	36.53 nm 0.273 −25.9 mV	Du et al., 2015
*Arthroderma fulvum*	Temperature, pH, concentration of AgNO_3_ and time of reaction	55°C pH 10 1.5 mM AgNO_3_ 12 h	20.56 nm PDI 0.27 Spherical	Xue et al., 2016
*Sclerotinia sclerotiorum*MTCC 8785	Culture media, quantity of biomass, concentration of AgNO_3_, pH and temperature	Potato dextrose broth 10 gm of biomass 2 mM AgNO_3_ pH 11 80°C	10–15 nm Spherical	Saxena et al., 2016
*Fusarium oxysporum*	Concentration of AgNO_3_ and quantity of biomass	5 mM AgNO_3_ 4.96 g/L of biomass	25–50 nm Almost spherical	Korbekandi et al., 2013
*Fusarium oxysporum*	Temperature culture media	28°C Modified medium for nitrate reductase	24 nm PDI 0.23 Spherical	Hamedi et al., 2017
*Rhizoctonia solani*	Culture media	Potato glucose broth	2–22 nm Spherical	Ashrafi et al., 2013
*Penicillium oxalicum* GRS-1	Temperature, quantity of biomassa, concentration of AgNO_3_ and pH	60°C 25 g of biomass 1.5 mM AgNO_3_ pH 7	10–40 nm Spherical	Rose et al., 2019

#### Effect of Temperature

The temperature used in the synthesis of silver nanoparticles employing fungi can affect parameters such as the speed of the synthesis and the size and stability of the nanoparticles (Elamawi et al., 2018).

In synthesis performed using the filtrate of *Trichoderma harzianum*, Ahluwalia et al. (2014) observed that the synthesis rate increased as the temperature was increased up to 40°C, which was considered the ideal temperature. In other work using the filtrate of *Fusarium oxysporum*, higher protein secretion by the fungal biomass was observed at temperatures between 60 and 80°C, with progressive increases of the synthesis rate and surface plasmon absorbance as the temperature increased (Birla et al., 2013). In synthesis using the endophytic fungus *Colleotrichum* sp. ALF2-6, Azmath et al. (2016) found that the reaction rate increased at higher temperatures, with the synthesis being completed within 20 min at temperatures above 50°C. In the work by Phanjom and Ahmed (2017), using *Aspergillus oryzae* (MTCC no. 1846), a higher temperature also increased the rate of synthesis, with temperatures of 30, 50, 70, and 90°C resulting in the synthesis being concluded in 6 h, 1 h, 45 min, and 20 min, respectively, while no synthesis occurred at 10°C. In the work of AbdelRahim et al. (2017), no synthesis of silver nanoparticles was observed using the filtrate of *Rhizopus stolonifer* at 80 or 10°C, which was attributed to denaturation or inactivation of enzymes and other molecules.

Although most studies have reported faster rates of synthesis at higher temperatures, it is important to take into account the quality of nanoparticles. In addition to influencing the synthesis rate, the temperature can affect nanoparticle size and stability. In the work of AbdelRahim et al. (2017), nanoparticles were obtained with sizes of 2.86, 25.89, and 48.43 nm, at temperatures of 40, 20, and 60°C, respectively, with the smallest size observed at the intermediate temperature. Shahzad et al. (2019) synthesized nanoparticles using the fungus *Aspergillus fumigatus* BTCB10, obtaining a size of 322.8 nm at 25°C and increasing size as the temperature was increased, reaching 1073.45 nm at 55°C. The size increase was attributed to the aggregation of nanoparticles at higher temperature. Elsewhere, Husseiny et al. (2015) used the fungus *Fusarium oxysporum* and found that the nanoparticle size decreased as the temperature was increased to 50°C, with the smallest size (30.24 nm) at this temperature. In the work by Fayaz et al. (2009), using the fungus *Trichoderma viride*, the nanoparticle size was also found to decrease as the synthesis temperature increased.

These different results indicate that the effect of temperature on the size and stability of the nanoparticles synthesized varies according to the fungus species used. Banu and Balasubramanian (2014a) reported that 30°C was the optimum temperature for production of high stability silver nanoparticles using *Isaria fumosorosea*. Balakumaran et al. (2015) also found that this was the optimum temperature for synthesis of nanoparticles using the fungus *Guignardia mangifera*. In the work of Costa Silva et al. (2017), using *Duddingtonia flagans*, the optimum synthesis temperature was 60 °C. These differences in the effect of the synthesis temperature occur even within the same fungal genus. *Trichoderma longibrachiatum* presented a specific synthesis temperature of 28°C, with no production of nanoparticles at 23 or 33°C (Elamawi et al., 2018), while *Trichoderma viride* presented viable synthesis at temperatures of 10, 27, and 40°C (Fayaz et al., 2009).

The occurrence of synthesis of nanoparticles by some fungal species at high temperatures indicates that electrons can be transferred from free amino acids to silver ions. However, very high temperatures, between 80 and 100°C, lead to denaturation of the proteins that compose the nanoparticle capping. This denaturation alters the nucleation of Ag^+^ ions, with the nanoparticles aggregating and increasing in size (Birla et al., 2013). According to Husseiny et al. (2015), unsuitable temperatures lead to increased nanoparticle size and loss of stability, due to the low activity of the enzymes involved in the synthesis.

#### Effect of pH

Adjustment of the synthesis pH can be used to control certain characteristics of the nanoparticles. Nayak et al. (2011) reported that the conformation of nitrate reductase enzymes could be altered according to the concentration of protons in the reaction medium, leading to alteration of the morphology and size of the nanoparticles. At higher pH, there is greater competition between protons and metal ions for establishing bonds with negatively charged regions, resulting in greater success of synthesis at alkaline pH (Sintubin et al., 2009).

Qian et al. (2013) observed that alkaline pH favored the synthesis of silver nanoparticles when AgNO_3_ was added to the filtrate of the fungus *Epicoccum nigrum*. Du et al. (2015) found that a more alkaline pH resulted in a shorter synthesis time and smaller nanoparticle size distribution and polydispersity index values. These characteristics indicate improved stability, due to the electrostatic repulsion of anions present in the dispersion (Gurunathan et al., 2009). Synthesis employing *Colleotrichum* sp. ALF2-6 at alkaline pH and a higher temperature of 50°C was faster than at lower pH and was completed in around 20 min (Azmath et al., 2016). In the synthesis performed by Birla et al. (2013), using *Fusarium oxysporum*, maximum nanoparticle production occurred between pH 9 and 11, with lower production at pH 7 and formation of aggregates between pH 3 and 5. However, Husseiny et al. (2015), using the same fungus, found that the nanoparticle synthesis rate decreased as the pH was increased, which was suggested to be due to lower activity of the reductases responsible for the synthesis at higher pH.

Some studies have reported successful syntheses at neutral or slightly alkaline pH. Nanoparticles synthesized using *Isaria fumosorosea* at pH 8.5 showed better physicochemical characteristics, compared to nanoparticles synthesized at pH 4.5 and 6.5 (Banu and Balasubramanian, 2014a). In synthesis using *Guignardia mangiferae*, no color change was observed between pH 1 and 4, while coloration started to appear at pH 5 and 6. As the pH was increased, the intensity of the dispersion increased, with the nanoparticles presenting greater monodispersion and stability at pH 7 (Balakumaran et al., 2015).

#### Effect of AgNO_3_ Concentration

In most of the studies employing fungi for extracellular synthesis of silver nanoparticles, AgNO_3_ was used at a concentration of 1 mM (Saxena et al., 2016; Xue et al., 2016). In some cases, a lower metal precursor concentration resulted in a smaller nanoparticle size and an improved dispersion (Kaviya et al., 2011; Phanjom and Ahmed, 2017). However, other studies obtained smaller sizes when intermediate AgNO_3_ concentrations were used. AbdelRahim et al. (2017), employing the fungus *Rhizopus stolonifer*, obtained the smallest nanoparticle size (2.86 nm) at 10 mM AgNO_3_, while sizes of 54.67 and 14.23 nm were obtained at 100 and 1 mM, respectively. Similar results were reported by Husseiny et al. (2015), using *Fusarium oxysporum*.

Phanjom and Ahmed (2017) studied the synthesis of nanoparticles using *Aspergillus oryzae* and different AgNO_3_ concentrations between 1 and 10 mM. It was observed that at AgNO_3_ concentrations up to 8 mM, the nanoparticles presented sizes between 7.22 and 17.06 nm, while the size increased to 45.93 and 62.12 nm at AgNO_3_ concentrations of 9 and 10 mM, respectively. This effect was attributed to the lack of functional groups available for the reaction when the metal precursor concentration was increased.

In addition to the effect on nanoparticle size, the AgNO_3_ concentration is related to the quantity of nanoparticles produced. In a study employing *Fusarium oxysporum*, it was found that the quantity of nanoparticles increased as the precursor concentration was increased between 0.1 and 1.5 mM, while no differences were observed at higher concentrations (Birla et al., 2013). Similar results were reported elsewhere for syntheses employing *Penicillium purpurogenum* (Nayak et al., 2011) and *Fusarium oxysporum* (Korbekandi et al., 2013).

These findings suggest that there is a limit to the concentration of AgNO_3_ used, in order to obtain nanoparticles with satisfactory physicochemical characteristics. The addition of excess amounts of metal ions results in very large nanoparticles with irregular morphology (AbdelRahim et al., 2017), due to competition between the silver ions and functional groups from the fungus filtrate (Shahzad et al., 2019). As the concentration of the metal precursor increases, so also does the intensity of color of the dispersion (Ahluwalia et al., 2014; Phanjom and Ahmed, 2017). In addition, a higher concentration of AgNO_3_ may lead to greater toxicity (Balakumaran et al., 2015).

#### Effect of the Culture Medium

It is known that microorganisms present different responses, depending on the culture medium and the cultivation conditions. Changes in these conditions result in the synthesis of different metabolites and proteins (Costa Silva et al., 2017).

In nanoparticle synthesis using fungi, a culture medium containing substrate specific for the enzymes that act in the synthesis can induce their production and release by the fungus, enhancing the reduction of silver and the formation of nanoparticles (Husseiny et al., 2015). In the work by Hamedi et al. (2017), *Fusarium oxysporum* was cultivated in a culture medium modified to induce nitrate reductase enzyme activity (0.35% yeast extract, 1% peptone, 0.35% potassium nitrate, and 1.5% glucose), as well as in malt glucose yeast peptone (MGYP) medium without enzyme induction (0.3% malt extract, 1% glucose, 0.3% yeast extract, and 0.5% peptone). The nanoparticle dispersions produced using the filtrate from the fungus cultivated in the enzyme induction medium presented higher concentrations and smaller sizes of the nanoparticles, which was attributed to stimulation of the enzymatic activity by the nitrogen source in the modified medium, hence increasing nanoparticle production.

Different behaviors were observed in studies in which different media were tested for the cultivation of fungi. Saxena et al. (2016) synthesized silver nanoparticles using *Sclerotinia sclerotiorum* cultivated in various broths, with the highest nanoparticle production achieved using potato dextrose medium. In work by Costa Silva et al. (2017), using the fungus *Duddingyonia flagans* for the synthesis of silver nanoparticles, the biomass was transferred to pure water and to water containing insect carapaces as a natural source of chitin (a substrate for fungal enzymes). The filtrate supplemented with chitin contained around three times more protein and presented higher nanoparticle production.

Birla et al. (2013) tested 10 different media for cultivation of *Fusarium oxysporum*, obtaining higher production of silver nanoparticles using the filtrate from the fungus cultivated in MGYP medium. Conversely, Ashrafi et al. (2013) found that the same medium inhibited the production of silver nanoparticles using the filtrate from *Rhizoctonia solani*, while the synthesis was successful when potato dextrose medium was used to cultivate the fungus. It was suggested that the activity of the enzyme responsible for the reduction process could have been inhibited by a component of the medium.

#### Effect of the Quantity of Biomass

The amount of biomass used can affect the synthesis and characteristics of silver nanoparticles. Some studies have reported higher nanoparticle production using lower biomass concentrations, while others have found higher synthesis rates using higher concentrations (Birla et al., 2013; Korbekandi et al., 2013; Balakumaran et al., 2015; Elamawi et al., 2018).

Balakumaran et al. (2015) used the filtrate obtained from 10, 20, and 30 g quantities of *Guignardia mangiferae* biomass in 100 mL of water, obtaining silver nanoparticles with better physicochemical characteristics when the lowest biomass concentration was used. Shahzad et al. (2019) evaluated the use of 1, 4, 7, and 10 g quantities of *Aspergillus fumigatus* BTCB10 biomass, observing greater production, smaller size, and better dispersion of the nanoparticles when the synthesis was based on the use of 7 g of biomass. In the work of Rose et al. (2019), employing *Penicillium oxalicum*, greater nanoparticle production was obtained using a higher biomass concentration, which was attributed to greater release of the nitrate reductase enzyme by the mycelium. Saxena et al. (2016) observed higher silver nanoparticle production when the amount of *Sclerotinia sclerotiorum* biomass was increased. Birla et al. (2013) reported the existence of a relationship between the amount of biomass and the release of biomolecules responsible for the synthesis.

Despite the differences in the amount of biomass used, depending on the fungus species employed, it can be concluded that successful synthesis of nanoparticles necessitates a suitable balance between the amount of organic material, derived from the fungus, and the amount of metal precursor (Phanjom and Ahmed, 2017; Shahzad et al., 2019).

In summary, it is clear that different synthesis conditions can result in different characteristics of the nanoparticles, as well as success or failure of the synthesis. However, the effects of the different parameters remain unclear, requiring further detailed studies for each organism used. It is also important to define the desired physicochemical characteristics of the nanoparticles, in order to establish the parameters used in the synthesis, such as temperature, pH, and time, among others. The optimization of synthesis techniques should enable the achievement of fast large-scale nanoparticle production. This opens avenues for the use of these nanomaterials to solve problems such as bacterial resistance to antibiotics and phytopathogens that affect agricultural production.

### Importance of Capping and Stabilization of the Nanoparticles

The synthesis of nanoparticles by non-biogenic methods requires an additional step in which polymers and surfactants are used to coat their surfaces. This process, known as functionalization, employs biomolecules that facilitate the anchoring of desired substances on the nanoparticle surfaces (Mout et al., 2012). In the case of biogenic synthesis, formation of the capping occurs simultaneously with formation of the nanoparticles, employing biomolecules derived from the organism used in the synthesis, so no additional steps are required (Chowdhury et al., 2014).

Biomolecules derived from the reducing organism have high capacities for binding to metals, with proteins and amino acid residues binding to the nanoparticle surfaces to form cappings that confer stability and prevent particle agglomeration and aggregation (Basavaraja et al., 2008; Gopinath et al., 2012). The binding of proteins at the surfaces, with consequent stabilization, may involve free amino groups or cysteine residues. Stabilization can also be provided by mycelial cell wall enzymes present in the filtrate, whose negative carboxyl groups provide electrostatic attraction (Gole et al., 2001; Husseiny et al., 2015). According to Gurunathan et al. (2009), the stability of silver nanoparticles is also provided by nucleophilic OH^−^ ions that are adsorbed on the surfaces, preventing aggregation and contributing to the synthesis of smaller nanoparticles by providing electrons for the reduction of silver ions.

**Figure 2:**
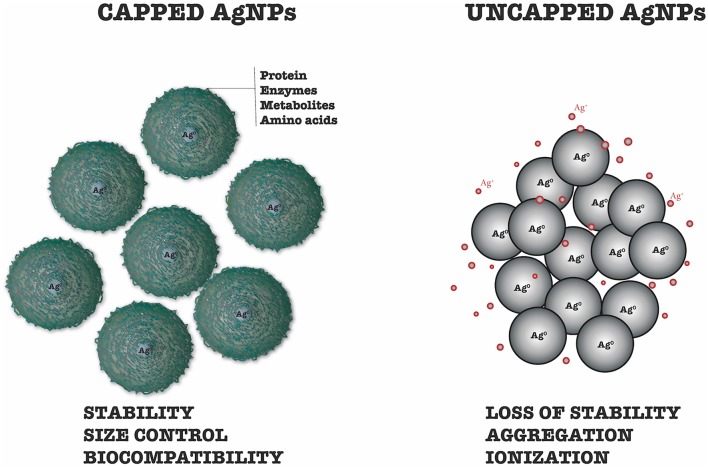
Differences between capped and uncapped silver nanoparticles.

In addition to conferring stability to the nanoparticles, the protein capping resulting from biogenic synthesis can act in the anchoring of drugs and genetic material for subsequent transport into cells (Hu et al., 2011; Zhang et al., 2016). The non-toxic organic composition of the capping means that it is biocompatible, which can increase the rate of internalization and retention of nanoparticles (Rodrigues et al., 2013; Mohanta et al., 2018; Figure 2).

Despite their importance, there have been few studies of the cappings on biogenic nanoparticles. Gade et al. (2008) detected sulfur atoms in samples of silver nanoparticles synthesized using *Aspergillus niger*, indicative of the presence of proteins enveloping the nanoparticles. Chowdhury et al. (2014) used scanning electron microscopy to detect the presence of cappings on biogenic silver nanoparticles. The nanoparticles were spherical, polydispersed, and were not in direct contact, even within aggregates, indicating good stability. The SDS-Page protein electrophoresis technique was employed to characterize the extracellular fungal proteins associated with the nanoparticles. Molecular weight bands between 50 and 116 kDa were attributed to proteins responsible for synthesis and stabilization of the nanoparticles. Both the filtrate and the capping removed from nanoparticles showed a band at 85 kDa, which corresponded to a protein that was suggested to be responsible for the stability of the nanoparticles.

Elgorban et al. (2016) synthesized biogenic silver nanoparticles and confirmed the presence of a capping by microscopic analyses. The use of transmission electron microscopy revealed a thin layer of organic material surrounding the particles. Signals corresponding to oxygen and carbon were observed using scanning electron microscopy, indicating the presence of organic compounds derived from the filtrate, which were adsorbed on the nanoparticle surfaces.

Devi and Joshi (2015) synthesized silver nanoparticles using the endophytic fungi *Aspergillus tamarii, Aspergillus niger*, and *Penicillium ochrochloron*. UV-Vis absorption analysis revealed peaks at 419, 430, and 430 nm, respectively. A peak at 280 nm was attributed to the presence in the filtrate of amino acid residues such as tryptophan and tyrosine, which were secreted by the fungi.

Given the importance of the cappings on biogenic nanoparticles, future studies are needed to investigate their compositions and biological activities. The use of synergy between nanometric silver and biomolecule cappings active against specific pathogens is likely to be a development in the near future.

## Applications

Silver nanoparticles synthesized using fungi have various potential applications in the areas of health, agriculture, and pest control. There are no reports concerning the better or worse activities of biogenic nanoparticles synthesized from different sources, such as fungi, bacteria, or plants. However, synthesis based on fungi may be advantageous in terms of production, due to the large quantities of metabolites produced. Another factor to consider is the capacity of fungi to produce antibiotics that could be contained in the capping and act in synergy with the nanoparticle core.

**Figure 3:**
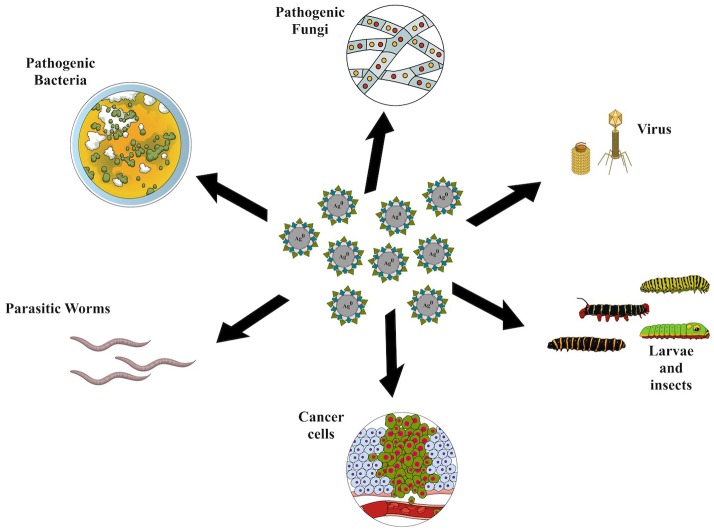
Applications of silver nanoparticles synthesized using fungi.

Many studies of biogenic synthesis of nanoparticles using fungi have shown results that are promising for the application of these systems in controlling pathogenic fungi and bacteria, combating cancer cells and viruses, and providing larvicidal and insecticidal activities (Figure 3).

### Health Applications

Several studies have described the use of biogenic silver nanoparticles for applications in the area of health, involving the control of bacteria and fungi. Bacterial growth is directly inhibited by the nanoparticles, which contact the cell wall and cause progressive metabolic responses, with the production of reactive oxygen species (Gudikandula et al., 2017).

The size of nanoparticles is one of the factors determining their antimicrobial potential, since smaller nanoparticles have greater effects (Lu et al., 2013). Small nanoparticles can penetrate the bacterial cell membrane and damage the respiratory chain, alter permeability, cause DNA and RNA damage, affect cell division, and lead to cell death (Morones et al., 2005; Rai et al., 2009). Nanoparticles also interact with the thiol groups of essential enzymes, releasing Ag^+^ ions that form complexes with nucleotides, damaging the microorganism DNA and inhibiting the activity of DNases (Li et al., 2010; Baker and Satish, 2012).

Materials with antifungal potential (such as biogenic silver nanoparticles) that are obtained from sustainable sources can be inexpensive and safe options for the treatment of systemic and surface fungal infections, enabling the control of resistant fungi (Ashajyothi et al., 2016). The large surface area of silver nanoparticles and the release of ions can contribute to high antimicrobial activity. The toxic ions bind to proteins containing sulfur, affecting cell permeability and leading to alteration of the DNA replication process. The inactivation of some enzymes is also caused by the binding of nanoparticles with thiol groups. This inactivation leads to oxidative stress, which influences electron transport and protein oxidation (Reidy et al., 2013; Rai et al., 2014; Fátima et al., 2016).

Table 2 shows some studies in which silver nanoparticles synthesized from different fungal species were employed for health issues.

**Table 2:** Health applications of silver nanoparticles synthesized by fungi.

**Synthesis source**	**Bioactivity**	**Target organism**	**Effective concentration**	**References**
*Macrophomina phaseolina*	Antibacterial	Multidrug resistant strains of *Escherichia coli*	0.51; 0.36; 0.25; 0.10; and 0.05 μg/mL (concentration-dependent)	Chowdhury et al., 2014
*Aspergillus tubingiensis and Bionectria ochroleuca*	Antibacterial	*Candida* sp. and *Pseudomonas aeruginosa*	0.11–1.75 μg/mL (*Candida* sp.) and 0.28 μg/mL (*P. aeruginosa*)	Rodrigues et al., 2013
*Rhizopus arrhizus IPT1011; Rhizopus arrhizus IPT1013; Trichoderma gamsii IPT853; Aspergillus niger IPT856*	Antibacterial	*E. coli, Staphylococcus aureus*, and *P. aeruginosa*	1.0; 5.0; 10; 50; and 100 μg/mL	Ottoni et al., 2017
*Fusarium verticillioides*	Antibacterial	*S. aureus* and *E. coli*	5 and 10 mM	Mekkawy et al., 2017
*Alternaria* sp.	Antibacterial	*Bacillus subtilis, S. aureus, E. Coli*, and *Serratia marcescens*	5, 10, 15, 20, and 25 mg/mL (concentration-dependent)	Singh et al., 2017
*Penicillium aculeatum Su1*	Antibacterial	*E. coli, P. aeruginosa, S. aureus, B. Subtilis*, and *Candida albicans*	50, 100, and 200 μg/mL (concentration-dependent)	Liang et al., 2017
*Pleurotus cornucopiae var. citrinopileatus*	Antifungal	*Candida* spp.	20, 40, and 60 mg/well	Owaid et al., 2015
*Inonotus obliquus*	Antibacterial and antiproliferative in cancer cells	*E. coli* KCTC 2441 A549 (lung cancer) and MCF-7 (breast cancer)	30 μL/disk	Nagajyothi et al., 2014
*Pleurotus djamor var. roseus*	Antiproliferative in cancer cells	PC3 cells (prostate cancer)	10–40 μg/mL	Raman et al., 2015
*Sclerotinia sclerotiorum MTCC8785*	Antibacterial	*E. coli* and *S. aureus*	100, 200, and 400 ppm	Saxena et al., 2016
*Colletotrichum* sp. *ALF2-6*	Antibacterial	*E. coli, Salmonella typhi, B. Subtilis*, and *S. aureus*	50 μg/mL (*S. aureus*) and 100 μg/mL (others)	Azmath et al., 2016
*Penicillium chrysogenum and Aspergillus oryzae*	Antifungal	*Trichophyton rubrum*	*P. chrysogenum* 0.5 μg/mL and *A. oryzae* >7.5 μg/mL	Pereira et al., 2014
*Schizophyllum radiatum*	Antibacterial	*B. subtilis* and *Salmonella paratyphi*	60 μL/well	Metuku et al., 2014
*Trichophyton rubrum, T. mentagrophytes and Microsporum canis*	Antifungal	*C. albicans*	4 μg/mL	Moazeni et al., 2012
*Aspergillus flavus*	Synergism with conventional antibiotics against multidrug-resistant bacteria	*Bacillus* spp., *Micrococus luteus, S. aureus, Enterococcus faecalis, E. coli, P. aeruginosa, Acinetobacter baumanii*, and *Krebsiella pneumoniae*	100 ppm	Naqvi et al., 2013
*Penicillium italicum*	Antibacterial and antifungal	Multidrug-resistant *S. aureus, Shewanella putrefaciens*, and *C. albicans*	25 μL/disk	Nayak et al., 2018
*Arthroderma fulvum*	Antifungal	*Candida* sp. and *Aspergillus* sp.	0.125–4.00 μg/mL	Xue et al., 2016
*Aspergillus versicolor*	Antibacterial	*S. aureus, Streptococcus pneumonia, P. Aeruginosa*, and *K. pneumoniae*	1 mg/mL	Netala et al., 2016
*Aspergillus terreus*	Antibacterial	*Salmonella typhi, S. Aureus*, and *E. coli*	11.43 μg/mL −308 μg/mL	Rani et al., 2017
*Fusarium oxysporum*	Antifungal	*Candida* spp. and *Cryptococcus* spp.	*Candida* spp. 0.84–1.68 μg/mL and *Cryptococcus* spp. 0.42–0.84 μg/mL	Ishida et al., 2014

Ahluwalia et al. (2014) synthesized silver nanoparticles using *Trichoderma harzianum*, which were used to control the bacteria *Staphylococcus aureus* and *Klebsiella pneumoniae in vitro*. The inhibition rates were concentration-dependent, with the gram-negative bacterium (*K. pneumoniae*) showing higher sensitivity. Balakumaran et al. (2015) reported the potential of silver nanoparticles synthesized using the fungus *Guignardia mangiferae* for the control of gram-negative bacteria, with effects including increased permeability, alteration of membrane transport, and release of nucleic acids.

The lower effects of silver nanoparticles toward gram-positive bacteria may be because the peptidoglycans that compose the cell wall act as a barrier that prevents internalization of the nanoparticles (Shrivastava et al., 2007). However, in some studies, the nanoparticles exhibited inhibitory effects against this bacterial type. In evaluation of the antimicrobial activity of silver nanoparticles synthesized using *Aspergillus niger*, Gade et al. (2008) observed inhibitory effects against the bacteria *E. coli* and *S. aureus* that were equivalent to those of the antibiotic gentamicin, with the gram-positive bacterium (*S. aureus*) showing higher sensitivity.

Silver nanoparticles have also been used in combination with antibiotics and antifungals, representing a possible solution to the problem of resistance toward these drugs used in the health area. Bhat et al. (2015) synthesized silver nanoparticles using *Candida albicans* and evaluated their effects when used alone or in combination with the antibiotic ciprofloxacin against *Staphylococcus aureus, Escherichia coli, Bacillus cereus, Vibrio cholerae*, and *Proteus vulgaris*. It was found that the activity of the antibiotic increased when it was used together with the nanoparticles, while the latter also showed antimicrobial potential when they were used alone.

Fátima et al. (2016) evaluated the antimicrobial and antifungal activities of silver nanoparticles synthesized using the filtrate from *Aspergillus flavus*. The nanoparticles were effective in controlling the bacteria *Bacillus cereus, Bacillus subtilis, Enterobacter aerogenes, Escherichia coli*, and *Staphylococcus aureus*, with *B. subtilis* and *E. coli* being most sensitive. The activity was concentration-dependent, with better results achieved using the nanoparticles in combination with the antibiotic tetracycline, rather than on their own. Concentration-dependent activity of the nanoparticles was also observed against the fungi *Aspergillus niger* and *Trichoderma harzianum*.

Gudikandula et al. (2017) used silver nanoparticles synthesized from the fungi *Trametes ljubarsky* and *Ganoderma enigmaticum* for the control of gram-positive and gram-negative bacteria (*Bacillus subtilis, Staphylococcus aureus, Micrococcus luteus, Bacillus cereus, Bacillus megaterium, Escherichia coli, Enterobacter aerogens, Klebsiella pneumoniae, Salmonella typhimurium, Proteus vulgaris, Pseudomonas aeruginosa*, and *Salmonella paratyphi*). Both types of nanoparticle were effective in controlling all the bacteria.

Ibrahim and Hassan (2016) synthesized silver nanoparticles using *Alternaria alternata*, which were capped with butyl acrylate and applied on cotton to inhibit the proliferation of microorganisms. At all the concentrations tested, the nanoparticle-treated cotton presented high antimicrobial activity against *E. coli* and *S. aureus*, achieving 99.9% inhibition. Biogenic silver nanoparticles can also be effective against resistant microorganisms. For example, Singh et al. (2014) used an optimized synthesis process employing *Penicillium* sp. to produce silver nanoparticles that showed potential for the control of multidrug-resistant *E. coli* and *S. aureus*.

In addition to their antimicrobial potential, biogenic silver nanoparticles can exert effects on tumor cells. Husseiny et al. (2015) evaluated the antibacterial and antitumor potential of silver nanoparticles synthesized using *Fusarium oxysporum*. The nanoparticles were effective in controlling *E. coli* and *S. aureus*, as well as a tumor cell line. A low IC50 value (121.23 μg cm3) for MCF-7 cells (human breast adenocarcinoma) was obtained following exposure of the cells to the nanoparticles, indicating high cytotoxicity and the potential for tumor control. The effect was attributed to the involvement of the silver nanoparticles in disruption of the mitochondrial respiratory chain, which led to the production of reactive oxygen species and hindered the synthesis of adenosine triphosphate (ATP), consequently damaging the nucleic acids (Husseiny et al., 2015).

Balakumaran et al. (2015) evaluated the cytotoxic potential of silver nanoparticles, synthesized using the fungus *Guignardia mangiferae*, against HeLa (human cervical carcinoma) and MCF-7 tumor cells, as well as normal Vero cells (African monkey kidney). Higher cytotoxicity was observed against the tumor cells, which showed signs of apoptosis, with condensed nuclei, membrane damage, and the presence of apoptotic bodies.

El-Sonbaty (2013) evaluated silver nanoparticles synthesized using the fungus *Agaricus bisporus* for their antitumor potential *in vitro* against MCF-7 tumor cells and *in vivo* against Ehrlich carcinoma in mice. The nanoparticles presented concentration-dependent activity in reducing the viability of the breast carcinoma cells. In the case of Ehrlich carcinoma, there was a decrease of blood vessels and an increase of apoptotic cells, with these effects being intensified when the application of nanoparticles was combined with exposure to gamma radiation. The cytotoxic effects of silver nanoparticles occur due to the interactions of the silver atoms with the groups of intracellular proteins and with the nitrogenous bases and phosphate groups of DNA (Sriram et al., 2010). Although the application of nanoparticles for the control of cancer is of considerable interest and has shown promising results in several studies, this technique still requires further investigation and the use of clinical trials (Balakumaran et al., 2015).

Biogenic silver nanoparticles have also shown effects against viruses. Gaikwad et al. (2013) synthesized silver nanoparticles using the fungi *Alternaria* sp., *Fusarium oxysporum, Curvularia* sp., *Chaetomium indicum*, and *Phoma* sp., which showed potential for reducing the replication of HSV-1, HSV-2, and HPIV-3 in cell cultures. The nanoparticles produced using *F. oxysporum, Curvularia* sp., and *C. indicum* were the most effective and presented low cytotoxicity, while the other two types were not used, due to their cytotoxicity.

### Agriculture and Pest Control Applications

There have been few studies to evaluate the potential of silver nanoparticles synthesized using biogenic methods for the control of phytopathogenic fungi in agriculture and pests. Table 3 shows some studies in which silver nanoparticles synthesized from different fungal species were employed in agriculture and pests control.

**Table 3:** Applications of silver nanoparticles synthesized by fungi in agriculture and pests control.

**Synthesis source**	**Bioactivity**	**Target organism**	**Effective concentration**	**References**
*Trichoderma harzianum*	Antifungal	*Sclerotinia sclerotiorum*	0.15 ×10^12^ and 0.31 ×10^12^ NPs/mL	Guilger et al., 2017
*Beauveria bassiana*	Insecticide	*Lipaphis erysimi*	(Concentration-dependent)	Kamil et al., 2017
*Trichoderma harzianum SYA.F4*	Antifungal	*Helminthosporium* sp*., Alternaria alternata, Phytophthora arenaria*, and *Botrytis* sp.	100 μg/mL	El-Moslamy et al., 2017
*Arthroderma fulvum*	Antifungal	*Fusarium* sp.	0.125–4.00 μg/mL	Xue et al., 2016
*Aspergillus versicolor*	Antifungal	*S. sclerotiorum* and *Botrytis cinerea*	150 ppm	Elgorban et al., 2016
*Epicoccum nigrum*	Antifungal	*Fusarium solani, Sporothrix schenckii, Cryptococcus neoformans, Aspergillus flavus*, and *Aspergillus fumigatus*	0.125–1.00 μg/mL	Qian et al., 2013
*Guignardia mangiferae*	Antifungal	*Colletotrichum* sp*., Rhizoctonia solani*, and *Curvularia lunata*	1 mg/mL	Balakumaran et al., 2015
*Fusarium solani*	Antifungal	Several fungal species isolated from wheat, barley and corn	1, 2, and 4%	Abd El-Aziz et al., 2015
*Alternaria alternata*	Antifungal combined with Fluconazol	*Phoma glomerata, Phoma herbarum*, and *Fusarium semitectum*	20 μL/disk	Gajbhiye et al., 2009
*Trichoderma harzianum*	Antiparasitic combined with Triclabendazol	*Fasciola hepatica*	50 μg/mL	Gherbawy et al., 2013
*Trichoderma harzianum*	Insecticide	*Aedes aegypti*	0.2–1.0%	Sundaravadivelan and Padmanabhan, 2014
*Isaria fumosorosea*	Insecticide	*Culex quinquefasciatus* and *Aedes aegypti*	0.3–1.0 ppm	Banu and Balasubramanian, 2014a
*Beauveria bassiana*	Insecticide	*Aedes aegypti*	0.06–1.0 ppm	Banu and Balasubramanian, 2014b

Elgorban et al. (2016) synthesized silver nanoparticles using the fungus *Aspergillus versicolor* and observed their effects against *Sclerotinia sclerotiorum* and *Botrytis cinerea* in strawberry plants. The nanoparticles showed concentration-dependent activity toward both pests, with the greatest effect against *B. cinerea*. Qian et al. (2013) synthesized silver nanoparticles using the fungus *Epicoccum nigrum* and observed their activity against isolates of the pathogenic fungi *C. albicans, Fusarium solani, Sporothrix schenckii, Cryptococcus neoformans, Aspergillus flavus*, and *Aspergillus fumigatus*. Balakumaran et al. (2015) synthesized silver nanoparticles using the fungus *Guignardia mangiferae* and reported their potential to control the phytopathogenic fungi *Colletotrichum* sp., *Rhizoctonia solani*, and *Curvularia lunata*. In other work, silver nanoparticles synthesized using the phytopathogenic fungus *Fusarium solani* isolated from wheat were shown to be effective for the treatment of wheat, barley, and maize seeds contaminated by different species of phytopathogenic fungi (Abd El-Aziz et al., 2015).

Several studies have investigated the combination of biogenic nanoparticles and conventional biocides. Gajbhiye et al. (2009) synthesized silver nanoparticles using the fungus *Alternaria alternata* and evaluated their potential, in combination with the antifungal compound fluconazole, against the phytopathogenic fungi *Phoma glomerata, Phoma herbarum*, and *Fusarium semitectum*, as well as the biological control agent *Trichoderma* sp. and the human pathogenic fungus *Candida albicans*. The combination of the nanoparticles and fluconazole was effective, with *C. albicans* showing the highest sensitivity after exposure, followed by *Trichoderma* sp. and *P. glomerata*. Potentiation of antifungal activity was not observed for *F. semitectum* or *P. herbarum*. Gherbawy et al. (2013) synthesized silver nanoparticles using *Trichoderma harzianum* and applied them in combination with triclabendazole for controlling the parasite *Fasciola* sp., which affects sheep and cattle. The nanoparticles combined with triclabendazole inhibited egg hatching by 90.6%, while use of the drug alone caused 70.6% inhibition. It was suggested that use of the nanoparticles together with the drug could be a way to overcome the resistance that the parasite has developed toward the drug.

Other studies have investigated the use of biogenic nanoparticles to control insect vectors. Sundaravadivelan and Padmanabhan (2014) synthesized silver nanoparticles using the filtrate from *Trichoderma harzianum* and observed concentration-dependent mortality when they were tested against the larvae and pupae of the dengue vector mosquito *Aedes aegypti*. In other work by Banu and Balasubramanian (2014a), silver nanoparticles synthesized using the entomopathogenic fungus *Isaria fumosorosea* were tested for control of the mosquito species *Culex quinquefasciatus* and *Aedes aegypti*, when applied between instars 1 and 4. Potential concentration-dependent control was observed for both species, with the greatest effectiveness against *Aedes aegypti*, for which the mortality of 1st instar larvae reached 100% within 24 h. The 4th instar larvae of both species showed lower susceptibility to the nanoparticles. Based on these results, these nanoparticles were considered as potential larvicides for mosquito control. The same authors synthesized silver nanoparticles using the mycelial extract of the entomopathogenic fungus *Beauveria bassiana* and obtained 100% mortality of the 1st and 2nd instar larvae of *Aedes aegypti* within 21 h of exposure to the nanoparticles. The authors concluded that use of the nanoparticles could be an environmentally safe strategy for vector control, following scale-up of production and field applications (Banu and Balasubramanian, 2014b).

## Conclusion

Recent studies show that the biogenic synthesis of silver nanoparticles using fungi offers several advantages and that these materials have promising potential for a range of applications in the areas of health and agriculture. The nanoparticles possess cappings derived from the fungi, which confer stability. Depending on the fungus used, this capping may also exhibit biological activity, acting in synergy with the effect of the nanoparticle core. The ability to use different species of fungi and to perform the synthesis under different conditions of temperature, pH, quantity of biomass, and concentration of the metal precursor, among others, enables the production of nanoparticles that have different physicochemical characteristics. However, in order to successfully use fungi for biogenic synthesis, there are a number of disadvantages that must be overcome. These include the need to know which fungus to use, its growth parameters, the need for sterile conditions, and the time required for fungal growth and for the synthesis to be completed. There can also be difficulties associated with scale-up, including the need for further investigation concerning the mechanisms of formation of capping layers and the molecules present in them.

Although progress is required on some issues, the studies published to date show that the use of fungi for biogenic synthesis of silver nanoparticles can lead to a wide range of possible applications. These nanoparticles offer considerable potential for exploitation in the control of pathogenic microorganisms.

## Author Contributions

MG-C and RL wrote the manuscript and prepared the figures. All authors read and approved the final manuscript.

### Conflict of Interest

The authors declare that the research was conducted in the absence of any commercial or financial relationships that could be construed as a potential conflict of interest.
